# Splenomegaly in Children and Adolescents

**DOI:** 10.3389/fped.2021.704635

**Published:** 2021-07-09

**Authors:** Meinolf Suttorp, Carl Friedrich Classen

**Affiliations:** ^1^Pediatric Hemato-Oncology, Medical Faculty, Technical University Dresden, Dresden, Germany; ^2^Division of Pediatric Oncology, Hematology and Palliative Medicine Section, Department of Pediatrics and Adolescent Medicine, University Medicine Rostock, Rostock, Germany

**Keywords:** splenomegaly, childhood, adolescence, pathophysiology, infections, infiltrative diseases, hematologic disorders, immunological disorders

## Abstract

In contrast to other lymphoid tissues making up the immune system, the spleen as its biggest organ is directly linked into the blood circulation. Beside its main task to filter out microorganism, proteins, and overaged or pathologically altered blood cells, also humoral and cellular immune responses are initiated in this organ. The spleen is not palpable during a physical examination in most but not all healthy patients. A correct diagnosis of splenomegaly in children and adolescents must take into account age-dependent size reference values. Ultrasound examination is nowadays used to measure the spleen size and to judge on reasons for morphological alterations in associated with an increase in organ size. An enormous amount of possible causes has to be put in consideration if splenomegaly is diagnosed. Among these are infectious agents, hematologic disorders, infiltrative diseases, hyperplasia of the white pulp, congestion, and changes in the composition and structure of the white pulp by immunologically mediated diseases. This review attempts to discuss a comprehensive list of differential diagnoses to be considered clinically in children and young adolescents.

## Introduction

Splenomegaly is not regarded as a disease of its own, but rather as a potential symptom associated with different disorders. In healthy individuals a spleen is usually not palpable in most cases. A patient exhibiting splenomegaly may therefore present with a number of clinical signs and laboratory or imaging findings that are commonly associated with distinct diseases ranging from self-limiting benign to infectious disorders or even malignancies. Thus, the finding of an enlarged spleen in a patient should be investigated properly to ascertain the etiology which may represent a diagnostic challenge in some cases.

**Figure 1:**
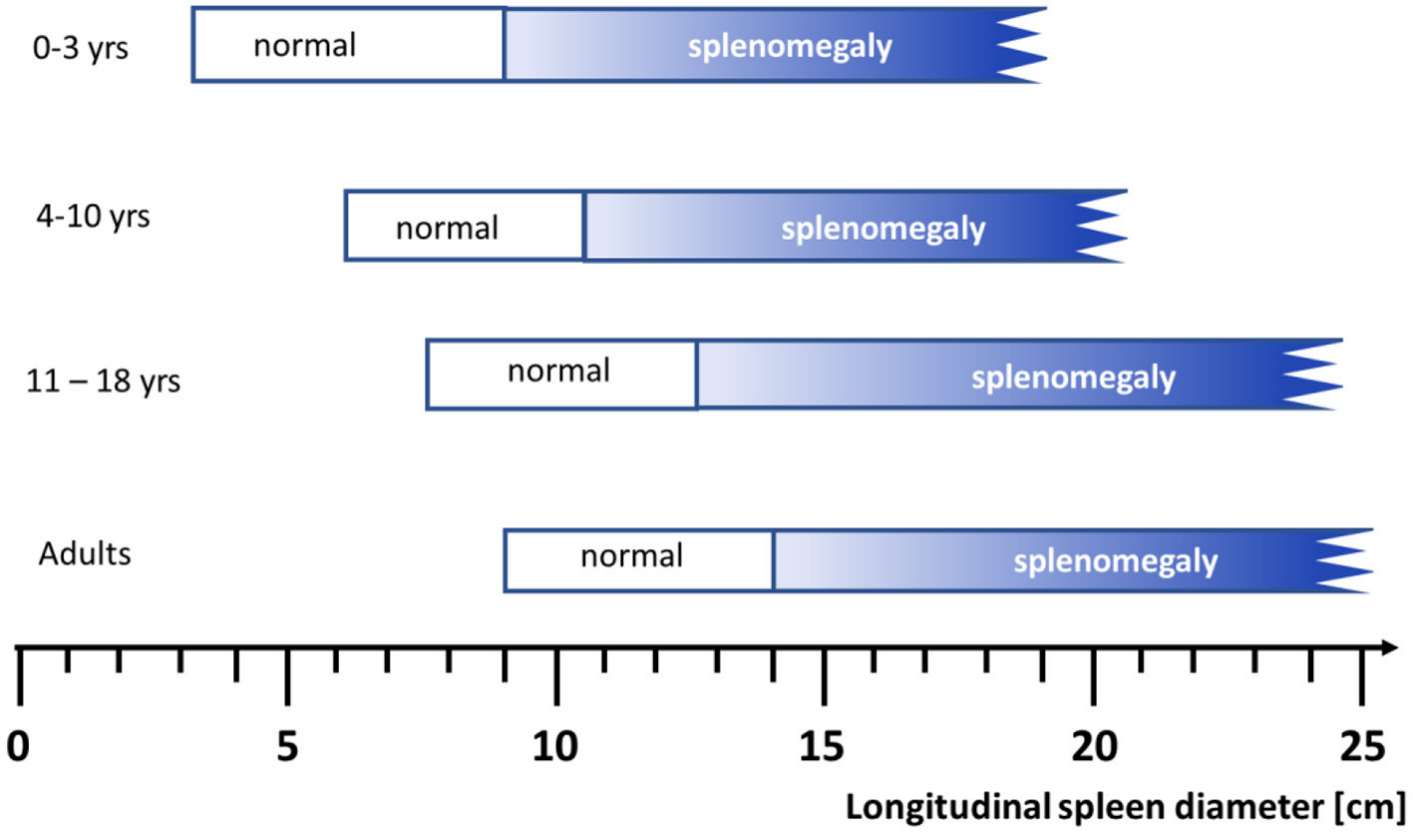
Range of spleen size (longitudinal diameter) assessed by ultrasound examination according to age in Caucasians (For details see **Table 2**, normal range denotes the 2.5th to 97.5th percentiles). Figure modified from (3).

Establishing a practical clinical definition of splenomegaly is not trivial and also there is no generally accepted grading of splenomegaly. Length or weight of the organ are typically used to characterize its size. In adults, spleens normally measure below 14 cm in the longest craniocaudal diameter, moderately enlarged spleens are 14–20 cm long, and severely enlarged spleens exceed 20 cm (1). Concerning weight, a spleen weighing 500–1,000 g is categorized as splenomegalic and more than 1,000 g are classified as “massive”. In adults, the clinical finding of a palpable spleen is generally considered as splenomegaly, but if imaging procedure are applied up to 16% of these organs are described to be of normal size (2). In children, any categorization must consider the age-dependent increase in size of the organ (3) (Figure 1).

This article aims to summarize the basic anatomy, the physiological background and the disorders causing splenomegaly. It may guide the pediatrician on the way to adopt a systematic approach to identify a serious disease including those challenging patients presenting with isolated splenomegaly.

## Anatomy and Physiology

The spleen is positioned in the upper left abdominal cavity between the gastric fundus and the diaphragm, adjacent to the costal margin between the 9th and 11th ribs (4). Ligaments fix the spleen in its normal position and absent or abnormal laxity of these suspensory may cause a so-called wandering spleen. This rare clinical entity predominantly affects children younger than 10 years with an incidence rate of 0.2% (5). If the spleen is attached only to a single elongated vascular pedicle, this situation predisposes to torsion and serious complications and is called a wandering spleen.

**Figure 2:**
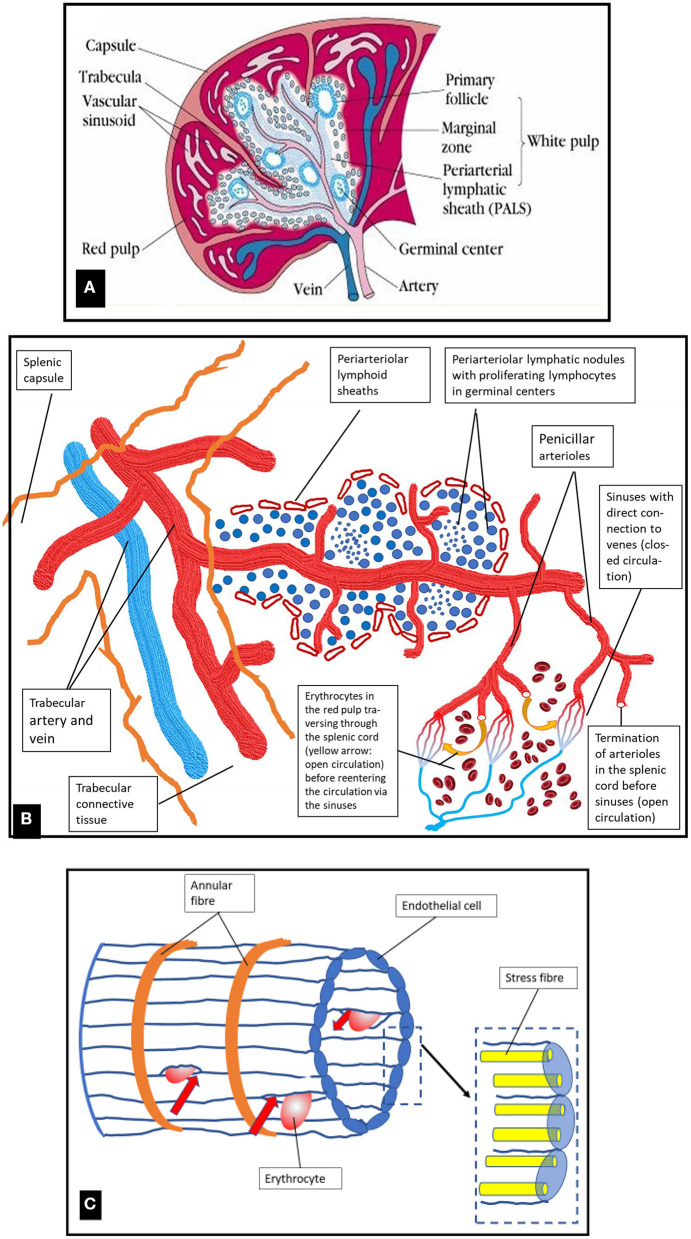
**(A)** Organization of macroscopic visible structures in the human spleen and schematic presentation of blood vessels and white and red pulp. Original photo from (6). **(B)** Schematic diagram of the microstructure of the spleen. For details see text. **(C)** Schematic drawing of a venous sinus located in the red pulp cords. Blood cells from the cords are only collected into the venous sinuses if they manage to pass the slits between the endothelial cells (shown by red arrows). Figure modified from (7).

The spleen represents the biggest organ of the lymphatic system with a spongy reddish-purple appearance due to its dense vascularization. The surface is made by a compact fibroelastic tissue capsule containing some smooth muscle fibers. The capsule protects the organ and contributes to its expansion and contraction. It is subdivided into many smaller internal sections termed lobules (Figure 2A). The parenchyma of the spleen is called pulp and contains two different types of tissues, termed white pulp (25% of the total splenic volume) and red pulp, each executing distinct physiological functions.

The white pulp -which is embedded in the red pulp- is primarily lymphatic and participates in the function of the immune system. The central arteries (intermediate-sized arterial vessels supplying each lobule) are surrounded by T- and B-lymphocytes forming aggregates, the periarteriolar lymphatic sheaths (T-cell dominant) and the follicles (B-cell dominant lymphatic nodules (Figures 2A,B). In the marginal zone separating white and red pulp, which is rich in macrophages and dendritic cells, pathogens, e.g., microorganisms and particulate antigens from the blood stream are filtered out and presented to the lymphocytes residing in the white pulp (4). Lymphocytes and antigen-presenting cells interact to built-up the humoral immune response via B-lymphocyte proliferation, and the innate immune response via cytokine signaling and phagocytosis (7). The spleen exerts a major role in the synthesis of immunoglobulin G, of properdin, which is an essential component of the alternate pathway of complement activation, and of the immunostimulatory tetrapeptide tuftsin (8).

The major proportion of the splenic volume (75%) is formed by the red pulp composed of a reticular connective tissue which incorporates splenic venous sinuses, cords, and perisinusoidal macrophages. The microstructure of the spleen is shown in a schematic diagram in Figure 2B. The initial segments of sinusoid capillaries are surrounded by special multicellular structures termed capillary sheaths. In humans these specialized vascular capillaries are located distal to branching points of terminal arterioles in the red pulp. Besides accumulations of B-lymphocytes they comprise specialized cuboidal inner sheath cells surrounded by macrophages (9, 10). Stromal sheath cells possibly play a role in enriching macrophages and B-lymphocytes at a location where antigens first enter the spleen, but their detailed specific role is a subject of ongoing research (11).

Approximately 5% of the cardiac output volume passes the spleen each minute (12). A 90% proportion of this blood volume travels to the red pulp (13). The human splenic microcirculation is both open and closed, with the open part forming the major proportion.

When the blood has left the open ends of the splenic red pulp arterioles (Figure 2B), it feeds an open microcirculation within the reticular connective tissue of the red pulp without endothelial barriers. The minor part of the closed microcirculation is formed by only a few connections between the red pulp capillary network and the red pulp venules (11).

From the open microcirculation in the red pulp the blood is re-collected for venous drainage. At this point for entering into the splenic venous capillaries, the blood flow has to slow down to allow cells to squeeze through the slits between the endothelial cells. Red cells with membrane alterations resulting in deformability impairment due to inherited membrane defects (spherocytosis, elliptocytosis) or hemoglobinopathies (sickle cell disease, thalassemia) and, in healthy individuals, senescent erythrocytes cannot pass through the slits (Figure 2C). Thus, this is the place where the degradation of old erythrocytes happens due to phagocytosis by red pulp macrophages. The venous sinuses are formed by a parallel lining of endothelial cells which are connected by stress fibers to annular fibers (Figure 2C). Contraction of the stress fibers will result in formation of slits between the endothelial cells. The width of theses slits regulates the passage of blood cells and blood plasma from the red pulp cords into the sinuses and further into the venous system (7).

## Epidemiology of Splenomegaly

Splenomegaly is a rare finding. In the USA the estimated prevalence is in the range of ~2% of the total population (14). The incidence of splenomegaly is strongly dependent on the geographical location reflecting the etiology as causes may vary with diseases prevalent in a given area (15). In Asia and Africa, tropical splenomegaly due to malaria, sickle cell disease or schistosomiasis is very common (16). Concerning the underlying causes of splenomegaly differences between developing and developed countries are quite obvious (17). Even between hospitals from different regions in the same country, the causes of splenomegaly can vary (18).

The spleen size may be influenced by variation between individuals and by different ethnicities and interfering genetic or infectious factors (Table 1). In Western world countries, the underlying diseases in all age groups causing splenomegaly are, in the order of decreasing frequency: hematological diseases, hepatic disease, infections, congestive or inflammatory diseases, and metabolic storage diseases (17, 18). In pediatric patients within the group of hematological disorders, the most common diagnoses associated with splenomegaly at diagnosis are acute leukemia, lymphoma, hemolytic anemia, chronic myeloid leukemia, and juvenile myelomonocytic leukemia.

**Table 1:** Causes of splenomegaly to be considered in individuals with different ethnicities due to interfering genetic or infectious factors (19).

	**Mediterranean ethnicity**	**African** **ethnicity**	**Asian** **ethnicity**	**Ashkenazi Jewish ethnicity**	**European or American-Amish ancestry**
Disorder with Increased incidence	Thalassemia Sickle cell anemia	Sickle cell anemia, Hereditary pyro-poikilocytosis Malaria	Portal hypertension secondary to non-cirrhotic portal fibrosis, Malaria	Gaucher disease, Niemann-Pick disease	Hereditary spherocytosis Pyruvate kinase deficiency

## Normal Range of the Spleen Size

An increase in the size of the spleen can be demonstrated by clinical examination (palpable spleen exceeding the left costal margin by more than 2 cm) and/or imaging procedures. Its median size corresponds approximately to an individual's fist (in adults 10–12 × 7–8 × 3–4 cm, weight 150–200 g, for children see Table 2). The longest craniocaudal diameter can easily be determined quantitatively by ultrasound. As a rule of thumb, from the age of toddlers onwards until puberty the formula

$$\begin{array}{l} \text{spleen length}\left[\text{cm}\right]=6\text{cm}+1/3\text{cm per year of age} \end{array}$$

is an applicable approach (20). However, as a word of caution, the spleen is palpable in 5–10% of all healthy children and in ~30% of all healthy neonates.

**Table 2:** Age dependent variation of spleen size parameters (volume, diameters, ratio of longitudinal spleen diameter to xiphod-pubic distance) as determined by ultrasound evaluation in *N* = 317 healthy children and adolescents with normal body weight and height (3–97% percentile) of both sexes and of Caucasian origin [For details see (3)].

**Variable**	**mean**	**SD**	**median**	**25th**	**75th**	**2.5th**	**97.5th**
**Volume (cm**^**3**^**)**							
0–18 years	81.0	51.4	70.8	41.2	105.8	13.2	221.2
0–3 years	33.1	15.5	31.9	23.5	37.9	9.3	68.3
4–10 years	74.9	30.1	70.2	54.8	90.2	30.1	147.4
11–18 years	125.5	52.1	111.2	90.5	153.0	42.0	264.6
**Longitudinal diameter (cm)**							
0–18 years	8.4	1.8	8.4	6.9	9.7	4.6	11.7
0–3 years	6.2	1.1	6.4	5.7	6.8	3.5	8.7
4–10 years	8.4	1.0	8.3	7.7	9.1	6.4	10.6
11–18 years	9.9	1.2	9.9	9.1	10.8	7.8	12.5
**Anterior-posterior diameter (cm)**							
0–18 years	3.2	0.8	3.2	2.6	3.7	1.7	4.9
0–3 years	2.4	0.5	2.3	2.1	2.7	1.4	3.4
4–10 years	3.2	0.6	3.2	2.8	3.5	2.0	4.5
11–18 years	3.8	0.7	3.8	3.4	4.2	2.6	6.0
**Ratio of longitudinal spleen diameterto**							
**xiphod-pubic distance (%)**							
0–18 years	35.8	5.5	35.5	32.6	38.8	25.7	47.4
0–3 years	37.8	5.7	37.2	34.3	41.4	27.1	51.9
4–10 years	35.2	5.9	35.2	31.8	37.7	25.5	52.1
11–18 years	35.0	4.3	34.4	32.1	37.9	26.2	45.2

Splenomegaly decreases in frequency with age because the ratio of the splenic volume to the abdominal volume declines over time (3). Most importantly, for establishing a correct diagnosis of splenomegaly, it is mandatory to have age- and body proportion-dependent normal values for the pediatric population from the corresponding geographic area (21–27). Assessment of splenic size should be done by ultrasound which has emerged during the last decades as the reliable method of choice and can be performed easily (3, 15, 28).

## Physiological Changes in Spleen Size

The spleen can contain ~8% of the total body erythrocytes which are densely packed, resulting in a high organ hematocrit around 80% (29). Changes in spleen size reflect reduced or increased adrenergic activity (30). At rest, when the oxygen transport capacity exceeds the body needs, the spleen exhibits a low-hematocrit, low-viscosity state, while during maximal exercise or apneic diving (stressed oxygen transport capacity) the spleen may decrease its volume by ~40%. Contraction seems to be an active response, mediated by alpha-adrenergic fibers in the splenic nerve (31), but also a passive collapse secondary to reduced blood flow (high hematocrit stage) has been discussed (32). During maximal exercise or apneic diving up to 50% of the blood stored in the spleen is transferred to the active circulation (33, 34). However, with regard to the relatively small size of the spleen in humans, the increase in the circulating total blood volume is <2% and the increase of hematocrit <10% (31). Thus, any effect upon physical performance is likely to be small.

During pregnancy, cardiovascular and hemodynamic changes occur, as the blood volume of the childbearing woman increases gradually over the 9 months of gestation, reaching a 40% increase by term (35). Closely related to this increase of plasma volume, the spleen increases in size by 50% following the equation: (36) spleen area [cm^2^] / BMI [kg/m^2^] = 1.598 + (0.032 × gestational age [weeks]).

## Pathological Enlargement of the Spleen Size

As there are multiple potential causes of splenomegaly, a careful and thorough evaluation is required and it may pose a challenge to find the underlying cause (8, 37). Situations of acute blood loss, therapeutic reduction of the hematocrit in relation to plasma volume (hemodilution), and infections all result in physiological transient enlargement of the spleen which is completely reversible after disappearance of the causing trigger (38). The underlying process can be classified according on the etiology and grouped into six major mechanisms:

i) infectious agents,
ii) hematologic disorders,
iii) infiltrative diseases,
iv) hyperplasia of the white pulp,
v) congestion, and
vi) immunologically mediated diseases.

These mechanisms will cause general splenomegaly. In addition, focal lesions (abscesses, cysts -either congenital or post-traumatic pseudocyst-, hemangioma, primary lymphoma in rare cases, metastasis) will not generally result in an overall increase of the size of the organ. However, focal lesions can be identified easily by ultrasound examination as typically only defined areas are of the organ are involved.

Hypersplenism (synonym: hypersplenic syndrome) must be distinguished from splenomegaly. (39) It is defined by a significant reduction in one or more blood cell types (erythrocytes, leukocytes, thrombocytes) in conjunction with splenomegaly and a compensatory increase of the corresponding precursor cells in the bone marrow. The main difference is that hypersplenism is a functional abnormality of the spleen while splenomegaly is a structural abnormality. Hypersplenism is a common manifestation in patients with portal hypertension (40). In addition, it is associated with chronic intravascular hemolysis, which may lead to platelet activation and thrombosis (see below).

**(i) Infections**

The spleen is the largest organ of the lymphatic tissue and the single lymphatic organ which is directly interposed into the blood circulation. During acute or chronic infections including viruses, bacteria, fungi, and mycobacteria (Table 3), the spleen performs enhanced work in antigen clearing and antibody production. This task is achieved by augmentation of the reticulo-endothelial cell number contained within the spleen and thus, these increase in immune functions may be accompanied by splenomegaly.

**Table 3:** Pathogenic mechanisms promoting splenomegaly in defined diseases and helpful further investigations to ascertain the diagnosis.

**Patho-mechanism**	**Disease**	**Further investigations**
Infections	EBV, CMV, bacterial sepsis, malaria, leishmaniosis, toxoplasmosis, bartonellosis, brucella, typhus, HIV, rickettsiosis, miliar tuberculosis, fungal infections	Fever? CRP? Specific serological antigen/antibody assessments, PCR as sensitive tool
	Abscess of the spleen or liver, cholangitis	Abdominal ultrasound
Hematologic disorders	Hemolytic anemias Erythrocyte membrane defects (e.g., spherocytosis) Hemoglobine defects (Sickle cell anemia, thalassemia) Autoimmune mechanisms	Complete blood count, microscopic examination of a peripheral blood smear, LDH, reticulocytosis, bilirubin,acid lysis test, Hb-electrophoresis
	Rhesus- and AB0-Eryhroblastosis	Only in neonates, Coombs test
Infiltrative diseases	Extramedullary blood cell formation (maximally stimulated hematopoiesis in thalassemia, as a consequence of the replacement of healthy hematopoiesis by infiltration of the bone marrow with malignant cells, myelofibrosis, osteopetrosis)	Hb-electrophoresis, bone marrow aspiration, bone marrow biopsy X-ray lower leg in osteopetrosis
	Leukemia (CML and JMML with slow progression)	Monitoring at fixed intervals
	Lymphoma, metastasis	Whole body MRI
	Storage diseases (M. Gaucher, M. Niemann-Pick, GM1-Gangliosidosis, M. Hunter, M. Hurler, Mucolipidosis)	Specific metabolites present in the urine and/or blood, activity of specific enzymes in leukocytes
Hyperplasia of splenic mono-cyte/macro-phage/histiocyte system	Sarcoidosis	Difficult, as no specific biomarker exists biopsy may be necessary
Congestion of blood flow	Portal hypertension (liver cirrhosis, liver fibrosis, multiple hepatic disorders, portal vein thrombosis, cavernoma)	Abdominal ultrasound examination
	Congestive heart failure	Echocardiography
Immunological mediated diseases	Systemic juvenile idiopathic arthritis (Morbus Still)	Joint inflammation, spiking fever, iridocyclitis
	Systemic lupus erythematodes (SLE)	Difficult, >4 out of 11 ACR criteria
	Systemic sclerosis (SS)	No single diagnostic test, diagnosis is usually based on clinical features and targeted investigations like nailfold capillaroscopy
	Wegener's disease	Antinuclear antibodies (ANA+)
	Polymyositis/dermatomyositis	Rule out malignancy in cases presenting with splenomegaly
	Mixed connective tissue disease (MCTD)	As listed for SLE, SS, and polymyositis
	Familal mediterranian fever (FMF)	CRP, Serum amyloid A, S-100-protein, mutations in the MEFV gene
	Autoimmune lymphoproliferative syndrome (ALPS)	“Double negative” CD3^+^CD4^−^CD8^−^ blood T-lymphocytes typically elevated (>5% virtually pathognomonic for ALPS)
	Hemophagocytic lymphohistiocytosis (HLH)	IL2-R, ferritin, serum triglycerides, blood cytopenia, elevated aminotransferase, coagulopathy
	Langerhans cell histiocytosis (LCH)	Biopsy of infiltrated organs, CD1a+ histio-cytes, mutation BRAFv600E in 50% of cases

In young adolescence, acute *infection with EBV* (mononucleosis, “student kissing disease”) is a very common cause of splenomegaly which usually is associated with a sore throat, fever and lymphadenopathy. Rare cases with resulting splenic rupture after a minimal trauma have been observed (41, 42).

Splenomegaly is often an impressive feature of *malarial infection*. While this parasitic infection is rarely observed in Western world countries, it represents a very common cause of splenomegaly worldwide. Repetitive bouts of malaria induce an abnormal immune response resulting in massive hyperreactive malarial splenomegaly (43). In addition, a large biomass of red cells infected with viable young and mature parasites accumulates in the spleen in asymptomatic persons chronically infected with malaria (44).

In *AIDS*, the spleen is commonly enlarged due to chronic viremia or opportunistic infections (45). Chronic *I.V. drug abuse* coincides with mild splenomegaly probably because on the basis of chronic, low-level sepsis from infections.

In the field of hematopoietic stem cell transplantation mobilization of stem cells from a healthy donors's bone marrow into the blood stream is achieved by a 5 day-long administration of *granulocyte colony stimulating factor (G-CSF)*. This scenario mimics bacterial sepsis as the serum levels of G-CSF reach a similar altitude. This treatment has also been reported to be associated with transient mild enlargement of the spleen (median increase in length 11 mm, range, 0–28 mm) in healthy adult donors (46). Data for children so far are missing.

**(ii) Benign hematologic diseases**

*Immune-mediated* destruction of erythrocytes (47), leukocytes, or platelets resulting in cytopenias (autoimmune hemolytic anemia, immune-mediated neutropenia, Felty syndrome, secondary immune thrombocytopenia but not in acute/primary immune thrombocytopenic purpura) may lead to splenomegaly. (48–50) Complete blood counts and careful microscopic examination of a blood smear are the first diagnostic steps which are followed by hemoglobin electrophoresis if thalassemia or sickle cell anemia are suspected. Among the hemolytic anemias not caused by defects of the hemoglobin synthesis *membrane disorders* like spherocytosis or elliptocytosis are the most common disorders which can be diagnosed non-specific by an acid lysis test or more specific in specialized laboratories for spherocytosis by eosin-5'-maleimide (EMA) fluorescent staining cytometry and for elliptocytosis by osmotic gradient ektacytometry (51–55).

Splenic sequestration crisis in pediatric *sickle cell disease* and in compound Hemoglobin S-beta-thalassemia plays a special role as up to 30% of these children may develop this life-threatening illness with a mortality rate of up to 15% (56). It is promoted by venous splenic vaso-occlusion by which a large proportion of the total blood volume becomes trapped within the spleen. This can result in a severe, rapid drop in the hemoglobin level leading to hypovolemic shock and possible death (57). The cascade of pathogenic events resulting in splenic sequestration crisis is still a matter of debate. Probably infectious conditions may promote sickle cell formation in the splenic red pulp. Reduced blood flow in parts of the cord, close to or within a draining vein is associated with a local decrease in oxygen concentration and will increase the formation of sickle cells. This scenario may be transient and reversible or result in extensive irreversible infarction. Consecutive multiple splenic infarcts will cause splenic fibrosis and scarring (58). Over time, this will lead in pediatric patients with sickle cell disease or compound Hb-S-beta-thalassemia to a small, auto infarcted spleen typically diagnosed in adolescent patients. Splenic sequestration crisis is rarely seen in adults because it only can occur in a functioning spleen. However, if splenic function is maintained, also late adolescent or adult patients may develop this type of crisis.

**(iii) Infiltrative diseases**

*Neoplastic cells* mainly comprising hematologic malignancies (Hodgkin- and Non-Hodgkin lymphoma, acute and chronic leukemias, myeloproliferative disorders) more or less regularly infiltrate the spleen causing splenomegaly (59). These diseases should especially be considered if constitutional symptoms and weight loss is complained. Asymptomatic splenomegaly may be the only physical finding in chronic myeloid leukemia in one third of pediatric patients (60). Abnormal peripheral blood smear and bone marrow examination or lymph node biopsy support key findings when making the diagnosis of malignant cell infiltration.

In case of bone marrow malfunction (e.g., fibrosis, infiltration by malignant cells) the spleen may resume in the postnatal life its embryonic role in blood cell formation. Beyond the fetal life this process termed *extramedullary hematopoiesis* must always be judged upon as a pathological finding. It causes the organ to increase in size substantially and may become extreme in myeloproliferative disorders (chronic myeloid leukemia, polycythemia vera, essential thrombocythemia, osteomyelofibrosis) or hematologic disorders (osteopetrosis, thalassemia) occurring at a very low incidence in the pediatric population (61). As pathological mechanisms, besides proliferation of hematopoietic cells *per se* also the sinusoids become occluded thus causing splenomegaly by blood afflux.

Among all infiltrative disorders causing splenomegaly *glycogen storage diseases* are observed rarely (62). If other more common causes are ruled out, this entity as listed in Table 3 should be considered in patients exhibiting clinical features consistent with inborn errors of metabolic storage diseases (63). Splenomegaly may present as the only symptom in some children and the identification of specific metabolites present in the urine and/or blood, or the reduced activity of specific enzymes in leukocytes will confirm a suspected diagnosis from this group of inherited diseases.

**(iv) Hyperplasia of the white pulp**

Activation and hyperplasia of the monocyte/macrophage system in the spleen is also known to play a causative role in splenomegaly. *Sarcoidosis* is a systemic inflammatory disease involving abnormal collections of inflammatory cells that form lumps in affected organs termed granulomas. The granulomas comprise tightly packed cell clusters with a central core of macrophages, epithelioid histiocytes, multinucleated giant cells, and unknown sarcoid antigens surrounded by a lymphocyte collar. Microscopic involvement of the spleen is present in ~75% of cases, but imaging procedures (CT, ultrasound, MRI) unravel innumerable small hypodense nodules only in about 5–10% of cases (64). Pediatric patients aged 8 to 15 years-old exhibit almost universal lung involvement, with mild splenomegaly. Additional organs like the eye, the skin, and the liver are involved in 30–40% of cases (65, 66). Children aged 5 years and younger only rarely exhibit splenic infiltration but typically show a triad of uveitis, arthropathy, and skin rash.

**(v) Congestion**

The close anatomical connection of the portal vein system with the splenic vein results in secondary splenic enlargement in case of blockade of the venous blood stream. Hypertension of the portal vein above normal (1–5 mm Hg) may occur due to increased intrahepatic vascular resistance (67). With a further increase in portal pressure above 10 mm Hg additional complications arise from the formation of portosystemic collaterals that can promote esophageal and gastric varices with a high risk of bleeding and mortality.

*Liver cirrhosis* and *schistosomiasis* are the leading causes for increased portal vein pressure worldwide. In addition, and with varying frequency on a given national background, *multiple hepatobilary disorders* may cause secondary splenomegaly (viral hepatitis, autoimmune hepatitis, cholangitis, choledocal cyst, biliary atresia, alpha-1-antitrypsine-deficiency, cystic fibrosis, M. Wilson, primary sclerosing cholangitis, galactosemia, Alagille syndrome, etc.). Abnormal findings during physical examination in conjunction with elevated liver enzymes and abnormal liver imaging are common cornerstones when establishing a diagnosis of liver disease. The resulting secondary splenomegaly is characterized histologically by an increased size of the red pulp with consecutive fibrosis and an accumulation of hemosiderin loaded macrophages. Siderotic splenic nodules corresponding to so-called Gamna-Gandy bodies detected by histological examination appear as punctate foci of low T1 and T2 signals in MRI (68, 69).

Thrombosis of the hepatic vein (*Budd Chiari syndrome*) may occur suddenly and thereafter the vein may or may not re-canalize (70). In patients with underlying liver diseases associated with increased resistance to portal flow re-canalization is observed less frequently. If the hepatic vein does not or only partially re-canalize, collateral veins dilate and become serpiginous. Variably these serpiginous vessels drain into the left and right portal veins or more distally into the liver. Additional connections may also exist with the pericholecystic veins. As sequela, happening variably within a time frame ranging from as short as a week to as long as a year, the normal single duct portal vein is replaced by numerous tortuous venous vessels appearing as cavernous transformation of the portal vein, which is also called portal cavernoma (71). The incidence of hypersplenism in conjunction with splenomegaly in patients with portal hypertension is high.

Congestive splenomegaly is a classic sign of organ congestion in decompensated *heart failure*. Insufficiency of the right ventricle, the left ventricle, or global heart insufficiency can be distinguished by echocardiography and may manifest as acute or chronic disease. The underlying causes are multiple and cannot be discussed in the context of this article. Congestive splenomegaly caused by cardiac insufficiency is usually accompanied by the clinical findings of decreased general performance, dyspnoea, lung edema, cardiac asthma, and peripheral edema. Patients with splenomegaly due to cardiac insufficiency must be treated by targeting at the underlying cause (72).

**(vi) Immunologically mediated diseases**

Connective tissue diseases may be associated with splenomegaly. These disorders in most cases are caused by autoimmune mechanism and include but are not limited to rheumatoid arthritis (RA), systemic lupus erythematosus (SLE), systemic sclerosis, granulomatosis with polyangiitis (GPA, formerly called Wegener's disease), polymyositis/dermatomyositis, and mixed connective tissue disease (MCTD).

A diagnosis of *rheumatoid arthritis* (73) or of juvenile idiopathic arthritis (JIA) which first appears before the age of 16 years is primarily made clinically and may be associated with splenomegaly. Systemic JIA (also termed *Still disease*) should be considered in pediatric patients with symptoms of arthritis, unexplained rash or prolonged fever -especially if quotidian-, iridocyclitis, generalized adenopathy, or splenomegaly. Macrophage activating syndrome (MAS—overlapping with HLH, see below) may be observed as a complication already at the onset of systemic JIA and in this situation splenomegaly can be found in more than half of the patients (74). The triad of rheumatoid arthritis, splenomegaly, and persistent neutropenia is termed *Felty's syndrome* (FS). Typically, FS is diagnosed at the age of 50–70 years and patients have had RA for more than 10 years. FS is observed very rarely in patients with JIA (75).

*Systemic lupus erythematosus* (SLE) can affect virtually any organ of the body and vascular changes are a hallmark in the pathogenesis. The clinical heterogeneity of systemic SLE and the lack of pathognomonic features or tests pose a diagnostic challenge for the clinician (76). The production of a number of antinuclear antibodies (ANA) are a prominent feature of the disease. Splenomegaly occurs in 9–18% of patients with systemic SLE and is more frequent in younger children (77).

*Systemic sclerosis* (SSc) is a disorder exhibiting a complex interaction of inflammation, fibrosis and vascular damage. Scleroderma and Raynaud's phenomenon are characteristic early findings. Progressive systemic sclerosis may be complicated by idiopathic portal hypertension, however, an enhanced resistivity of the splenic artery may be associated with systemic SLE. High values of the splenic artery resistivity index (SARI) at doppler ultrasound assessment point to an intrinsic spleen vascular damage allowing to separate the cause of splenomegaly from liver fibrosis (78).

Granulomatosis with polyangiitis (GPA, formerly called *Wegener's disease*) is a form of necrotizing vasculitis that is associated with granuloma formation. Nose, lungs, kidneys are primarily affected. Reports of splenic involvements are rare. In some patients with GPA and splenomegaly infarctions of the distal parenchymal splenic arteries have been observed with either a focal or diffuse pattern (79).

*Polymyositis* is characterized by inflammation and degeneration of the muscles. When the skin is also affected, it is called *dermatomyositis (DMS)*. Approx. one third of adult patients with DMS develop cancer and in a considerable proportion (~40%) the diagnosis of DMS is made after the diagnosis of a malignancy. The proportion of children with malignancies is smaller but careful evaluation to rule out a malignoma -especially in cases presenting with splenomegaly- should be undertaken at the time when a diagnosis of DMS is established (80).

*Mixed connective tissue disease* (MCTD), also called Sharp syndrome, is a rare autoimmune disorder. MCTD is characterized by findings commonly observed in the following three different connective tissue disorders: systemic lupus erythematosus, scleroderma, and polymyositis. Some patients affected may also show symptoms of rheumatoid arthritis. MCTD commonly affects women under the age of 30 years. Splenomegaly can be observed as described above in cases exhibiting the three disorders.

*Familial Mediterranean fever* (FMF) is a hereditary auto-inflammatory disorder caused by mutations in the Mediterranean fever gene, which encodes the pyrin protein (81). It is characterized by periodic episodes of fever and serosal inflammation, generally lasting 1–3 days and spontaneous remission. At preschool age, fever may be the single symptom during a febrile attack but in more than 90% of patients also abdominal pain affects the whole abdomen with all signs of peritonitis. Gross amounts of serum amyloid A protein (SAA) are produced during attacks, and at a lower rate in between (82). Amyloid A accumulates mainly in the kidney, as well as the heart, spleen, thyroid and gastrointestinal tract. Mild splenomegaly is a finding in younger adults (83). In one half of the children with FMF splenomegaly is detectable by ultrasound (84). The increase in size of the spleen seems to be higher during attacks compared to attack-free periods (85). In animal models, macrophages located in the vicinity of splenic amyloid deposits are considered to play a role in amyloid degradation (86).

*Autoimmune lymphoproliferative syndrome* (ALPS) is a rare lymphoproliferative disorder, usually presenting in pediatric patients with splenomegaly, massive lymphadenopathy, and in the course of the disease with an increased incidence of lymphoma. It represents a genetically dysregulated immune condition of abnormally prolonged lymphocyte survival caused by defective Fas mediated apoptosis (87). Double negative T-cells (TCRαβ^+^ CD4^−^ CD8^−^ T cells) are a characteristic finding in ALPS. During periods of disease activity these cells infiltrate and disorganize the splenic marginal zone and cause abnormal marginal zone B-cell function (88). Most patients will require treatment with immunosuppressive drugs that will effectively reduce or ameliorate symptoms (89).

*Hemophagocytic lymphohistiocytosis* (HLH) is a rare but severe form of immune dysregulation. The disorder presents as unremitting fever, cytopenia, hepatosplenomegaly, coagulopathy, and elevation of typical HLH biomarkers among these ferritin, IL2-R, serum triglycerides, blood cytopenia, elevated aminotransferase (90). Cytohistological examination of lymphoid tissue, bone marrow and spleen show accumulation of lymphocytes and macrophages, sometimes with hemophagocytic activity (91). HLH may cause a life-threatening state of hyperactivated immune response that is observed in the setting of genetic mutations (familial HLH) as well as infectious, inflammatory, or neoplastic triggers (92). Familial HLH is treated with chemotherapy for bridging the time until hematopoietic stem cell transplantation can be performed. HLH occurring in the context of rheumatic diseases (macrophage activation syndrome) is treated with glucocorticoids, IL-1 blockade, or cyclosporine A. In other forms of HLH, addressing the underlying trigger is essential.

The frequency of occurrence of *histiocytic diseases* peaks in childhood and adolescence. Disorders belonging to this entity are generally rare and their variable clinical course and variable morphology contribute to the diagnostic challenge. Histiocytoses are subdivided into Langerhans cell histiocytosis (LCH) and the so-called non-LCH, such as juvenile xanthogranuloma, Erdheim-Chester disease and Rosai-Dorfman disease (93). In childhood the most common forms of histiocytosis comprise LCH (also called histiocytosis X) and juvenile xanthogranuloma. LCH primarily affects children from birth to age 15 years (94). The disease exhibits features of both an abnormal reactive and a neoplastic process with an abnormal increase in proliferating dendritic histiocyte cells. These cells may infiltrate a single organ whereas disseminated LCH may involve multiple organs like bone marrow, lungs, liver, spleen, lymph nodes, gastrointestinal tract, and the pituitary gland. Based on the extent of organ involvement at diagnosis, namely, single-system LCH, and multisystem LCH with unifocal or multifocal organ involvement must be distinguished. Organs may be affected by infiltration only or / and by resulting dysfunction (95). Diagnosis requires a biopsy and histiocytes in LCH are CD1a+ while BRAFv600E mutations have been found in 50–55% of cases. The survival rates for patients without organ dysfunction are excellent, however, mortality rates for patients with organ dysfunction may reach 20% (96). Splenomegaly in patients with LCH usually is part of a multifocal systemic disease, whereas isolated splenic LCH is extremely rare.

## Concluding Remarks

The spleen combines in one organ an efficient phagocytosis of senescent red cells in conjunction with the recycling of iron, the cognition, capture and elimination of pathogens, and the induction of adaptive immune responses. The separation into different splenic compartments promotes tasks that are not fulfilled in other lymphoid organs.

Splenomegaly in most cases is the result of a systemic disease. The underlying pathophysiological mechanism can be subdivided into infectious, hematologic, infiltrative, vascular, and immunological diseases with resulting abnormalities of the lymphoid, reticuloendothelial, or vascular components of the spleen. A precise assessment of spleen dimensions is easily and reliably achievable by ultrasound examination. The interpretation of the data must be based on age-dependent normal values in pediatric patients.

**Figure 3:**
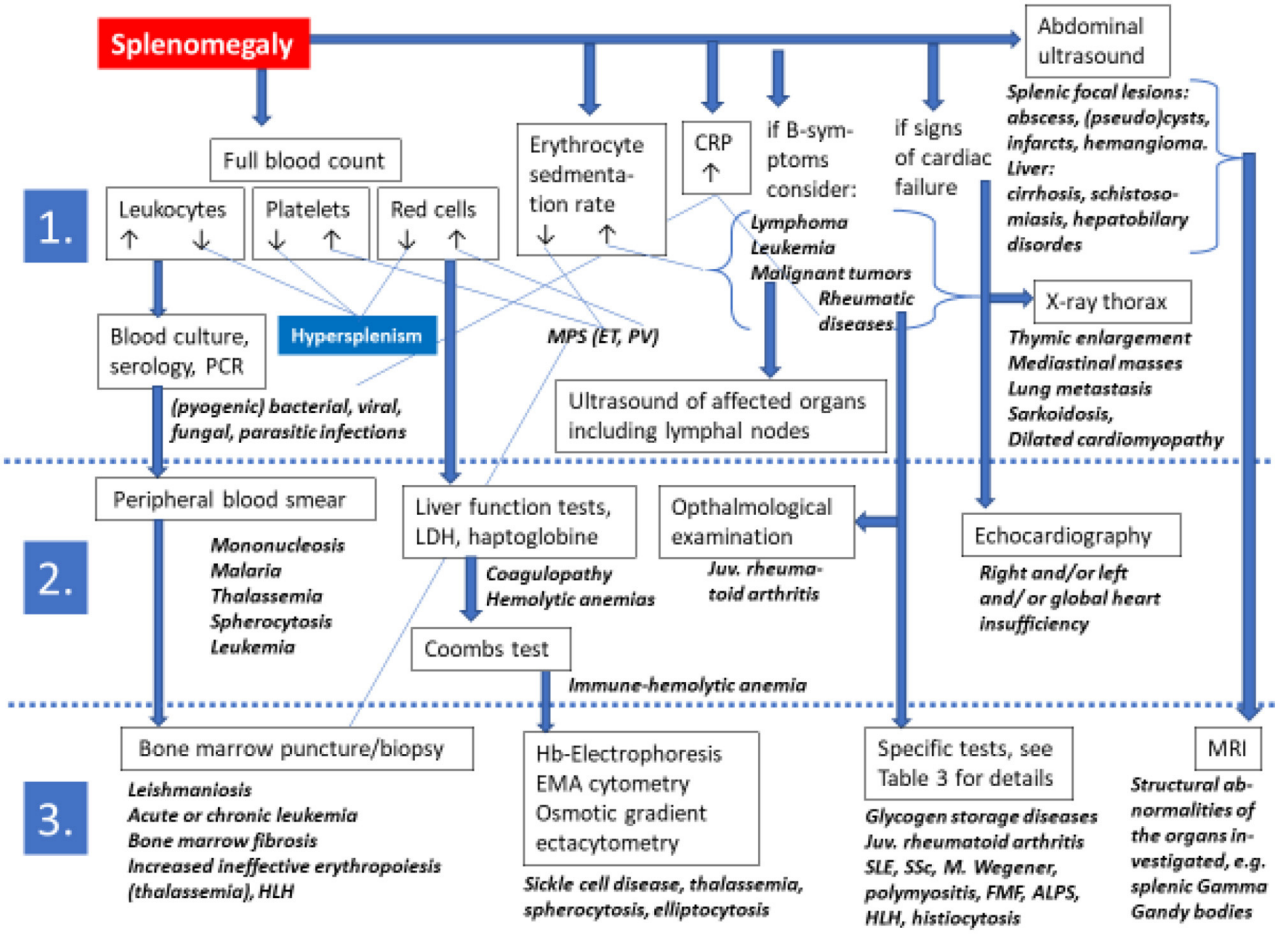
Algorithms for a non-evidence based diagnostic approach toward a pediatric or adolescent patient presenting with splenomegaly on physical examination. Diagnostic steps are grouped into 1st line, 2nd line, and 3rd line approaches. The extensive list of differential diagnoses excludes more detailed information in this flow chart for which the reader is kindly referred to the text and to Table 3 of this review. ALPS, autoimmune lymphoproliferative syndrome; CRP C, reactive protein; EMA, eosin-5'-maleimide; FMF, familial Mediterranean fever; Hb, hemoglobin; HLH, hemophagocytic lymphohistiocytosis; juv., juvenile; LDH, lactate dehydrogenase; MRI, magnetic resonance imaging; PCR, polymerase chain reaction; SLE, systemic lupus erythematosus; SSc, systemic sclerosis.

The list of possibilities included in the differential diagnosis of splenomegaly in children and adolescents is extensive. Thus, the assessment of an enlarged spleen hinges on a comprehensive analysis of clinical data. Essential is the correct classification of the associated findings especially of the full blood count, and the status of the liver and lymph nodes (Figure 3). In a given patient the clinician must carefully balance all possible differential diagnoses, the individual clinical features and the results of laboratory and imaging investigations.

Infection is the most common cause of an enlarged spleen in children, but after treating an infection successfully the size of the spleen must be re-evaluated and persisting splenomegaly should always be taken seriously. The individual clinical picture guides the urgency of diagnostic procedures. Beyond acute viral illness, constitutional symptoms such as fever, weight loss or night sweats are symptoms suggesting a disease present in other organs or systems. But also isolated splenomegaly can be associated with malignancy.

So far there is no established evidence-based management strategy for a pediatric patient with splenomegaly, however, essential investigations must exclude lymphadenopathy, pathological findings of the liver, gut, and chest (Figure 3) (97). In selected patients with only mild splenomegaly it may be appropriate to monitor the course for some weeks. However, if the size of the spleen enlarges and/or new symptoms or clinical signs arise, then the patient should be reassessed and establishing a diagnosis becomes mandatory.

## Author Contributions

MS and CC developed the concept of this review, wrote the first draft of the typoscript, critically discussed the content, and approved the final version of the typoscript.

## Conflict of Interest

The authors declare that the research was conducted in the absence of any commercial or financial relationships that could be construed as a potential conflict of interest.
